# Reproductive Success Beyond Pollinators: Microhabitat Effects and Pollen Dynamics in *Epipactis bugacensis*, a Traditionally Obligately Autogamous Orchid

**DOI:** 10.3390/plants15050709

**Published:** 2026-02-26

**Authors:** János György Nagy, Anna Morzsányi, Adrián Molnár, István Somogyi, Melinda Molnár, Miklós Sárospataki, Gábor Lőrinczi, Kamilla Nagy, Lilla Diána Gilián

**Affiliations:** 1 Sustainability Competence Center, Széchenyi István University, 9026 Győr, Hungary; 2 Institute of Horticultural Sciences, Hungarian University of Agriculture and Life Sciences, 2100 Gödöllő, Hungary; morzsanyianna@gmail.com; 3 Institute of Agronomy, Hungarian University of Agriculture and Life Sciences, 2100 Gödöllő, Hungary; adrinmol@gmail.com; 4 Kiskunság National Park Directorate, 6000 Kecskemét, Hungary; somogyii@knp.hu; 5 Department of Rural and Regional Development, Hungarian University of Agriculture and Life Sciences, 2100 Gödöllő, Hungary; melindamolnar@yahoo.com; 6 Department of Zoology and Ecology, Hungarian University of Agriculture and Life Sciences, 2100 Gödöllő, Hungary; sarospataki.miklos@uni-mate.hu; 7 Department of Ecology, University of Szeged, 6726 Szeged, Hungary; lorinczig@gmail.com; 8 Department of Obstetrics and Gynecology, Semmelweis University, 1085 Budapest, Hungary; kamillanagy19@gmail.com; 9 Faculty of Humanities, Eötvös Loránd University, 1088 Budapest, Hungary; gilianlilla@gmail.com

**Keywords:** autogamy, geitonogamy, Halictidae, orchid pollination, xenogamy

## Abstract

Orchid pollination is traditionally considered to rely on intact pollinarium transfer by animal vectors. Species lacking a functional viscidium are generally classified as obligately autogamous. In this study, we investigated the reproductive biology of *Epipactis bugacensis*, a taxon long regarded as strictly self-pollinating. Floral visitor activity was assessed through repeated field observations, and pollinator dependence was tested using a pollinator-exclusion (net-covering) experiment at two Hungarian populations, combined with measurements of fruit set, capsule volume, seed number, and seed density. We documented a previously unreported pollen-transfer mechanism in *E. bugacensis*, whereby halictid bees fragment pollinia and transfer these fragments in their scopa to neighboring flowers enabling geitonogamous deposition and suggesting the potential for xenogamous pollen transfer. Other visitor taxa showed no evidence of effective pollen transport. Mesh coverage increased fruit set, capsule volume, and seed number, while seed density remained unchanged. Reproductive output declined from basal to apical positions along flowering shoots, revealing strong internal resource-allocation constraints. Overall, *E. bugacensis* is predominantly self-pollinating but not strictly obligate autogamous, and its reproductive success is governed primarily by microhabitat quality rather than pollinator availability.

## 1. Introduction

Since Darwin’s epoch-making work, numerous detailed and comprehensive studies have been published on the pollination strategies of species in the Orchidaceae family [1,2,3,4,5,6,7,8]. These works reveal that the taxa of this extremely species-rich family (29,524 accepted species [9]) employ a wide variety of strategies to ensure their sexual reproduction. In all but 185 species belonging to the subfamilies Apostasoidae and Cypripedoidae [9], pollen grains are aggregated into pollinia, which, together with a stalk and a viscidium, form a pollinarium [10,11]. According to the available literature, pollinia break away from the flower as a whole and are transported by sticking to the pollinator’s body with the help of their sticky pad or disc (viscidium). Since the pollinator usually visits several flowers with the pollinium stuck to its body, it can fertilize one or more flowers, depending on whether the entire pollinium is transferred to the stigma of the visited flower (e.g., *Orchis mascula*, *Orchis morio*, *Anacamptis morio*, *Dactylorhiza romana* [12], *Oncidium* spp. [13]) or whether only pollen tetrads or massules breaking off from the pollinium are transferred [14,15]. Researchers on the subject consider it a fact that pollination of the remaining three subfamilies is only possible by removing the entire pollinarium (e.g., [3,4,5,6,8]). The rare exception to this is a Japanese publication, which discusses the role of thrips in the pollination of *Epipactis thunbergii*, where tiny insects carried the fragments of granular pollinarium stuck to their bodies [16].

Pollen and nectar produced by angiosperms are the main attractants for their pollinators, as these are vital food resources for them [17]. Nectar primarily provides carbohydrates, while pollen serves as the main source of proteins, but it also contains carbohydrates, lipids, and a range of vitamins and mineral nutrients [18]. Nectar, or the promise of it, is a major attractant for most food-rewarding or food-deceptive orchids [19]. Notable exceptions include species of Newlandia and Apostasia, which offer pollen as a reward [20]. In addition, some species from other subfamilies such as *Cypripedium wardii* [21] and *Gastrodia elata* [22] employ pseudopollen that combines reward and deception to attract both flies and bees as pollinators.

*Epipactis* species belonging to the subfamily Epidendroideae were described as having nectar flowers too [23,24,25]. The genus has a Holarctic-Paleotropical distribution, and contains a complex of allogamous and autogamous species, between which the species boundaries are not always easy to define [26,27,28]. As of the latest update, GBIF lists 82 accepted species and 39 accepted hybrids among them [29]. This number is much higher than the most conservative molecular estimates, which recognize 11 species [27], but is consistent with broader taxonomic treatments that include many morphologically distinct or regionally described species [28,30].

The high genetic similarity between *Epipactis bugacensis* examined in this study and the three other taxa, *Epipactis tynensis, Epipactis dunensis*, and *Epipactis rhodanensis*, none of which have been reported in Hungary, supports the possible treatment of these four taxa as a single species [27]. Of these, only two are currently accepted as distinct species: *E. bugacensis* and *E. dunensis* [31,32]. The obligately autogamous *E. rhodanensis* is regarded as a synonym of the similarly obligately autogamous *E. bugacensis*, whereas the facultatively autogamous *E. tynensis* is treated as a synonym of the facultatively autogamous *E. dunensis* [28,29,31,32]. In this study, we follow the taxonomic treatment of *E. bugacensis* as a distinct species according to Plants of the World Online [31,32]. Accordingly, all references to *E. bugacensis* in this study pertain to the populations examined within the Carpathian Basin.

Whether an *Epipactis* species is considered autogamous or allogamous is determined based on the anatomical characteristics of its flowers [33]. In autogamous species, the rostellum is reduced or absent, the viscidium is absent or non-functional, the clinandrium is underdeveloped, and the pollinia are easily disintegrated, crumbly, and projected over the pistil. This allows pollen to come into direct contact with the pistil and facilitates self-pollination [34,35].

Hungarian *Epipactis* species were also categorized as autogamous or allogamous based on the morphology of their gynostemium and, more specifically, the presence or absence of a sticky pad and its functionality [19]. If a species lacks a viscidium (e.g., *E. albensis*, *E. müllerii*, *E. placentenia*, *E. leptochila*) or its flowers do not open (*E. futakii*), then the species is considered obligate autogamous. Species in which the viscidium is present but dries out a few hours after the flower opens and becomes non-functional (e.g., *E. voethii*, *E. neglecta*, *E. futakii*, *E. nordeinorum*, *E. bugacensis*, *E. tallosii*, *E. moravica*, *E. mecsekensis*, *E. exilis*, *E. pontica*) [27,36,37,38,39,40,41,42] are considered obligate autogamous as well. In these cases, as pollen transport has been considered impossible, the formation of hybrids has been considered only conceivable if a pollinator arriving with the pollinium of an allogamous species pollinates the autogamous flower [33].

At the same time, it is known that regardless of the morphology of the column, the hypochile of the lip (labellum) of all *Epipactis* species in Hungary contains nectar [19] and that the pollinator attraction is influenced not only by nectar but also by floral scent, color and shape [6,43,44,45,46,47]. Across this genus, pollination is primarily mediated by insects such as bees, wasps, hoverflies, mosquitoes, and beetles, with species-specific variation depending on habitat and floral traits [48,49,50,51,52,53,54]. This apparent contradiction between the assumed impossibility of pollen transport in obligately autogamous species and the continued investment in pollinator-attracting traits raises fundamental questions regarding the functional role of flower-visiting organisms in these taxa [54].

In this study, we examined whether any flower-visiting animals can transport pollen to or from the flowers of *E. bugacensis*, which is described as an obligately autogamous species. If so, we further aimed to identify the taxa involved and the mechanisms by which pollen transfer occurs. In addition, we assessed whether isolation from potential pollinators affects the following traits by comparing pollinator-isolated and pollinator-exposed individuals with respect to (i) fruit set, (ii) capsule volume, (iii) seed number per capsule, (iv) seed density within capsules, and (v) plant mortality.

## 2. Results

### 2.1. Flower Visitor Observations

Flower visitors of *E. bugacensis* recorded during field observations are summarized in Table 1.

Detailed behavioral descriptions of the most frequent or ecologically relevant visitors are provided below.

**Halictidae**

**Figure 1:**
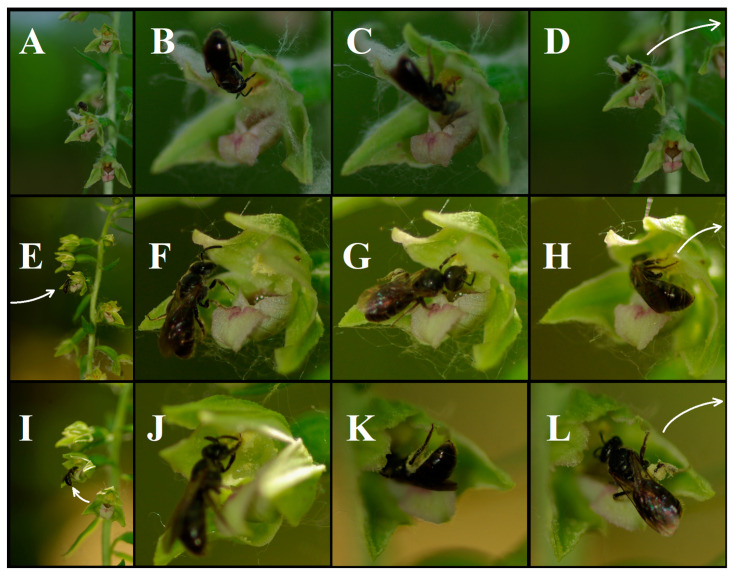
Behavior of a halictid bee on the flowers of *E. bugacensis*. (**A**–**D**) Brief inspection of an empty flower, followed by immediate departure without foraging. (**E**–**H**) Disaggregation of the pollinium and consumption of its fragments. (**I**–**L**) Disaggregation of the pollinium and transport of pollinium fragments. Pollinium fragments adhere to the pistil of the visited flower and the pistil of a neighboring flower, and may also be transferred to the pistils of other individuals. Arrows show the movement of the bee from flower to flower. Photographs taken by János György Nagy on 4 June 2018.

A sweat bee arrived at a flower at 10:36 on 4 June 2018 with seemingly completely pollen-free body hairs. It landed on the abaxial (outer) surface of the upper sepal (Figure 1A) and then moved onto its adaxial surface, facing the interior of the flower. The individual examined the gynostemium and the hypochil (Figure 1B) and, after finding both structures empty (Figure 1C), departed within the same minute (Figure 1D).

At 11:06, another (or possibly the same) individual arrived at a different flower on another plant, with the upper flowers still in bud and the lower ones just beginning to open (Figure 1E). The bee, already carrying some pollen on its legs, landed on the lip and on the left sepal when viewed from the front (Figure 1F) and immediately began chewing and scraping the pollinia using its mandibles and first pair of legs (Figure 1G). The bee attempted to access the pollinia from multiple angles, circling its body around the gynostemium several times while its pollen-bearing hairs repeatedly contacted the stigma surface. During the approximately two minutes spent on this flower, it consumed most of the pollen and some of the scraped pollinium fragments scattered across the inner surface of the flower, primarily onto the stigma, while additional fragments adhered to its pollen-transporting hairs (Figure 1H). The bee then flew upward to the next flower (Figure 1I).

The bee also disaggregated the pollinium at the second flower as well. During this process, it frequently used its legs to transfer fragmented, sticky pollinium material to the hind-leg scopa, which consequently bore visibly increased amounts of fragmented pollinium material (Figure 1K). Meanwhile, part of the crushed pollinium material was also deposited onto the stigma and other floral structures (Figure 1J). The individual repeatedly changed its position on the flower—its pollen-bearing body hairs often contacted the stigma—and departed only after completely fragmenting and removing both pollinia (Figure 1L).

**Culicidae**

A single culicid mosquito landed on the lip and remained motionless at the boundary between the epichil and mesochil, with its mouthparts inserted into the hypochil. No contact with the gynostemium was observed.

**Syrphidae**

***Episyrphus balteatus*:**

**Figure 2:**
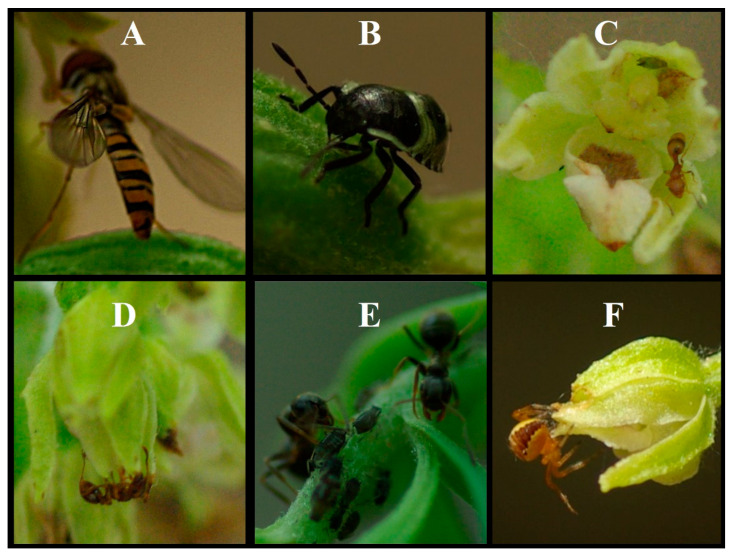
Other visitors of *E. bugacensis*. (**A**) *Episyrphus balteatus*. (**B**) A pentatomid nymph. (**C**) A *Temnothorax unifasciatus* worker and an aphid on the inner surface of the sepal. (**D**) A *Myrmica* sp. worker. (**E**) Workers of *Lasius* sp. and aphids. (**F**) *Araneus diadematus*. Photographs by János György Nagy ((**A**,**B**,**F**); 23 June 2022), Adrián Molnár ((**C**); 20 June 2019), and János György Nagy ((**D**,**E**); 27 June 2022).

Individuals repeatedly approached the flowers and briefly landed near them before immediately taking off again (Figure 2A). They frequently hovered around the inflorescences, but no approach to or contact with either the hypochil or the gynostemium was observed.

***Platycheirus splendidus*:**

Individuals flew systematically from plant to plant, hovering in front of the flowers, and inspecting them individually. No physical contact with the flowers was observed.

**Pentatomidae**

In 2019, pentatomid bugs were observed resting on leaves and at the base of flowers without any detectable activity. In 2022, one individual was observed feeding on a developing capsule (Figure 2B).

**Aphididae**

Aphids occurred in large numbers primarily on the inflorescences of bud-stage plants. They appeared to be tended by ants and were also observed on the inner surfaces of the petals (Figure 2C,E).

**Formicidae**

***Temnothorax unifasciatus:***

Individuals moved over the entire plant and visited all flowers. Inside the flowers, they explored extensively, crowded into the hypochil, remained on the lip and near the pollinarium, and tended aphids feeding on the sepals. Pollinia were completely absent from flowers in which these ants were present (Figure 2C).

***Myrmica* sp.:**

Individuals moved actively on the plants and inside the flowers, visiting both the hypochil and the gynostemium (Figure 2D).

***Lasius* sp.:**

Individuals moved over the entire plant and tended aphids feeding on bud-stage plants (Figure 2E). They were not observed on flowering individuals.

**Araneae**

Spiders constructed webs among the flowers and waited for prey while sitting on the plants.

***Araneus diadematus*:**

An individual initiated web construction at the apex of a flower (Figure 2F). No contact with the gynostemium was observed.

### 2.2. Changes in Different Sexual Propagation Traits in Covered and Uncovered E. bugacensis Individuals

#### 2.2.1. Effect of Net Covering on Fruit Set at the Jászfényszaru (JF) Site

At the JF site, flower-to-capsule conversion differed markedly between covered and uncovered treatments. Covered individuals produced capsules from 62 out of 116 flowers (53.4%), whereas uncovered individuals formed capsules from only 150 out of 679 flowers (22.1%).

Fisher’s exact test revealed a highly significant difference between treatments (odds ratio = 4.05, 95% CI: 2.69–6.09, *p* < 0.001), indicating that net-covered plants were more than four times as likely to develop capsules from flowers than uncovered individuals. A complementary binomial GLMM accounting for flower nesting within plants (plant random intercept) supported the same conclusion (covered vs. uncovered OR = 6.01, 95% CI: 2.99–12.06).

#### 2.2.2. Effect of Net Covering on Reproductive Success Traits of *E. bugacensis* Across Four Treatments (JF Covered, JF Uncovered, Harkakötöny (HK) Covered, HK Uncovered)

Treatments at the JF site generally exhibited higher capsule volumes and seed numbers compared to the HK site, particularly under covered conditions, while seed density showed no significant differences among groups. Because capsule traits were measured on multiple capsules per plant, we additionally repeated group comparisons using plant-level aggregated values (mean of the two lowest capsules per individual) as independent units. This plant-level re-analysis supported the same qualitative pattern: capsule volume and seed number were highest at the JF site (especially in covered plants), whereas seed density remained broadly similar across site × treatment groups.

##### Capsule Volume

**Figure 3:**
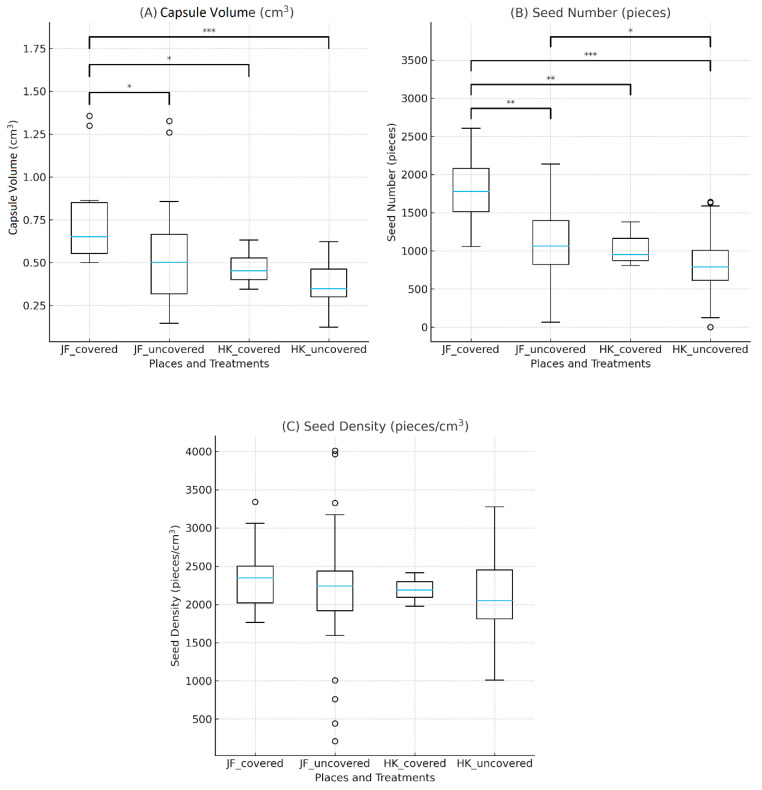
Capsule volume (**A**), seed number (**B**), and seed density (**C**) of *E. bugacensis* across four treatments (JF covered, JF uncovered, HK covered, HK uncovered). Boxplots display median, interquartile range, and extreme values; circles represent outliers. Significant pairwise differences were evaluated using Dunn’s post hoc test with Holm correction following a Kruskal–Wallis analysis. Asterisks represent significance levels (*p* < 0.05: *; *p* < 0.01: **; *p* < 0.001: ***).

Capsule volume differed significantly among the four site-treatment groups of *E. bugacensis* (Kruskal–Wallis test: H = 22.81, *p* < 0.001). Post hoc comparisons using Dunn’s test with Holm correction showed that the JF covered group produced significantly larger capsules than all other groups (*p* < 0.05 for all pairwise contrasts), with large effect sizes (Cliff’s delta δ = 0.51–0.85) (Figure 3A). No significant difference was found between the JF uncovered and (HK) covered groups, while the HK uncovered group consistently exhibited the smallest capsule volumes.

Capsule volume ranged from 0.1466 to 1.3565 cm^3^, with an average of 0.4735 cm^3^ for all samples.

##### Seed Number

Seed number also varied markedly across treatments (H = 26.80, *p* < 0.001). Similar to capsule volume, the JF covered plants produced the highest number of seeds, significantly exceeding all other groups after Holm correction (*p* < 0.05) (Figure 3B). Effect sizes were predominantly large (δ = 0.64–0.90), indicating strong biological relevance. The difference between JF uncovered and HK covered was negligible, consistent with a lack of statistical significance.

In total, 191,632 seeds were counted from 196 capsules across the two study sites, highlighting the robustness of the dataset. Of these, 129 capsules collected at the JF site contained 134,852 seeds, while 67 capsules from the HK site contained 56,780 seeds. Seed number per capsule ranged from 0 to 2606, with a mean of 978 seeds per capsule (or 1009 when excluding six capsules that contained only abortive seeds). Notably, five of the six capsules containing exclusively abortive seeds originated from uncovered individuals.

##### Seed Density

In contrast to capsule volume and seed number, seed density did not differ significantly among the four groups (H = 2.57, *p *= 0.463) (Figure 3C). All pairwise comparisons were non-significant after correction, and effect sizes were small or negligible (Cliff’s δ = 0.04–0.28).

Mean seed density values were highly comparable between localities and treatments, averaging 2399 seeds cm^−3^ in JF covered plants, 2181 seeds cm^−3^ in JF uncovered plants, 2191 seeds cm^−3^ in HK covered plants, and 2132 seeds cm^−3^ in HK uncovered plants; they ranged from 210 to 4011 seeds cm^−3^, with an average of 2177 seeds cm^−3^ for all samples. These similar values show that seed density remained stable regardless of locality or net-covering treatment.

#### 2.2.3. Changes in Reproductive Success Traits from the Basal to the Apical Capsules in Covered and Uncovered *E. bugacensis* Individuals at the JF Site

At the individual plant level, linear regressions revealed spatial patterns in seed number, capsule volume, and seed density along the flowering shoot, with differences between covered and uncovered treatments.

In complementary mixed-effects analyses accounting for repeated capsule measurements within plants, capsule position showed an overall negative association with seed number and capsule volume at the population level, while between-plant variability in slope magnitude was observed. No consistent population-level association was detected between capsule position and seed density.

##### Seed Number

**Figure 4:**
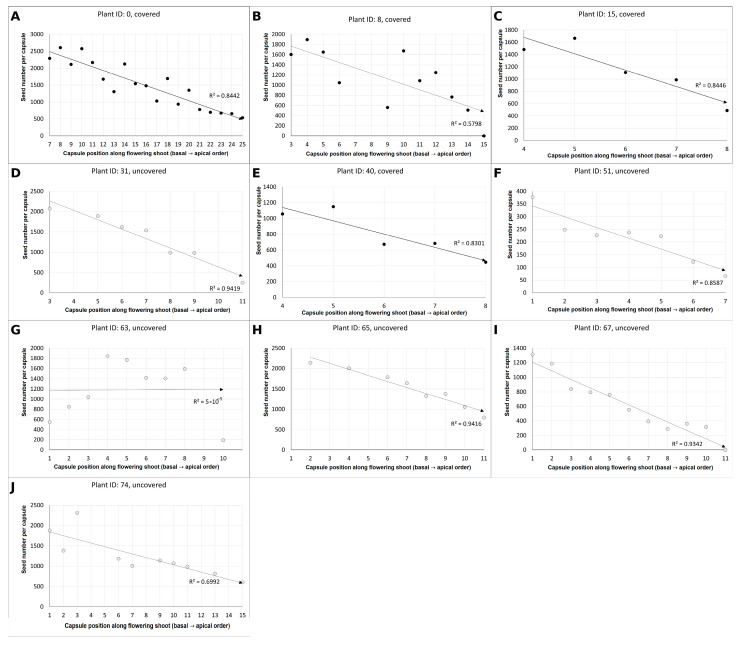
Pearson correlation between seed number and capsule position along the flowering shoot (from the lowest to the uppermost capsule) in covered and uncovered *E. bugacensis* individuals at the JF site. Panels (**A**–**J**) represent individual plants (Plant ID indicated in each panel). Black dots indicate capsules of covered individuals, while grey dots indicate capsules of uncovered individuals. Solid lines represent fitted linear regressions with corresponding coefficients of determination (R^2^). *x*-axis: Ordinal position of capsule-producing flowers along the flowering shoot toward the apex; *y*-axis: Seed number per capsule.

In covered and uncovered plants, seed number per capsule declined from basal toward apical positions along the flowering shoot, as indicated by consistently negative regression slopes (Figure 4). Strong linear relationships (*p* < 0.01) were detected in two covered (A, B) and five uncovered (D, F, H, I, J) individuals, whereas weak (0.01 < *p* < 0.05) correlations occurred in two covered individuals (C, E), with non-significant (*p* ≥ 0.05) correlations for one uncovered (G) plant.

##### Capsule Volume

**Figure 5:**
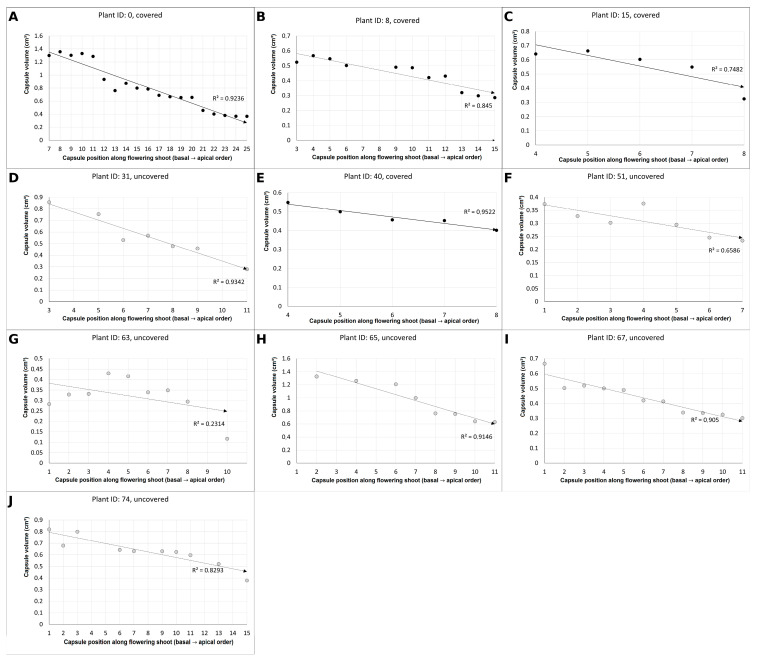
Pearson correlation between capsule volume and capsule position along the flowering shoot (from the lowest to the uppermost capsule) in covered and uncovered *E. bugacensis* individuals at the JF site. Panels (**A**–**J**) represent individual plants (Plant ID indicated in each panel). Black dots indicate capsules of covered individuals, while grey dots indicate capsules of uncovered individuals. Solid lines represent fitted linear regressions with corresponding coefficients of determination (R^2^). *x*-axis: Ordinal position of capsule-producing flowers along the shoot toward the apex; *y*-axis: Capsule volume (cm^3^).

Capsule volume also decreased from basal toward apical positions (Figure 5). Strong linear relationships (*p* < 0.01) were detected in three covered (A, B, E) and four uncovered (D, H, I, J) individuals, whereas weak (0.01 < *p* < 0.05) correlations were found in one uncovered (F) individual, and non-significant (*p* ≥ 0.05) correlations occurred in one covered (C) and one uncovered (G) plant.

##### Seed Density

**Figure 6:**
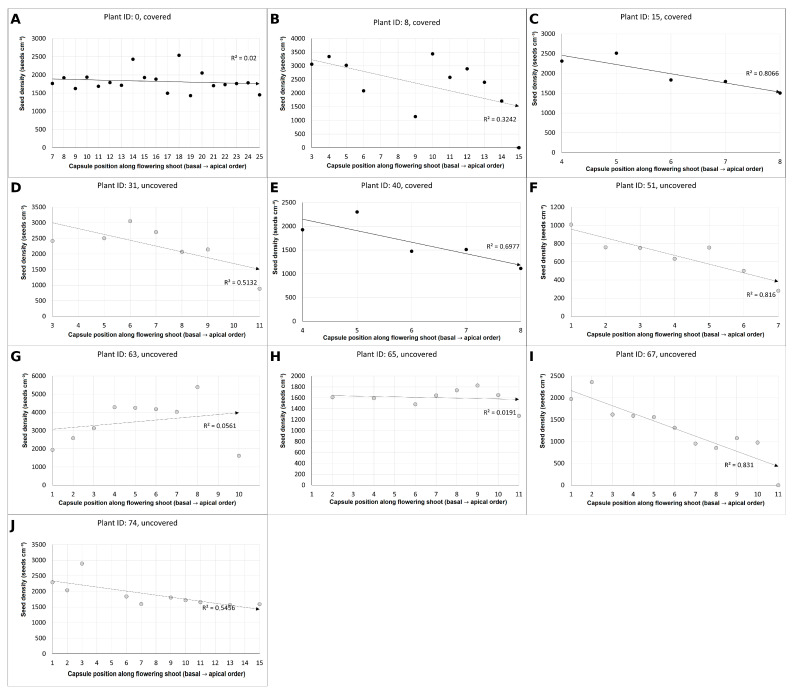
Pearson correlation between seed density and capsule position along the flowering shoot (from the lowest to the uppermost capsule) in covered and uncovered *E. bugacensis* individuals at the JF site. Panels (**A**–**J**) represent individual plants (Plant ID indicated in each panel). Black dots indicate capsules of covered individuals, while grey dots indicate capsules of uncovered individuals. Solid lines represent fitted linear regressions with corresponding coefficients of determination (R^2^). *x*-axis: Ordinal position of capsule-producing flowers along the shoot toward the apex; *y*-axis: Seed density (seed number/cm^3^).

Seed density exhibited the weakest spatial structuring (Figure 6). Strong linear relationships (*p* < 0.01) were detected only in two uncovered (F, I) individuals, whereas weak (0.01 < *p* < 0.05) correlations occurred in two covered (B, C) and two uncovered (G, J) individuals, with non-significant (*p* ≥ 0.05) correlations in two covered (A, E) and two uncovered (D, H) plants.

### 2.3. Mortality Outcomes

Mortality rates differed in magnitude between the two treatment groups at the Jászfényszaru site: 45.5% in the covered group and 75.8% in the uncovered group (Table 2). Although the uncovered plants exhibited a notably higher mortality rate, Fisher’s Exact Test indicated that this difference was only marginally significant (*p* = 0.0664). The analysis was based on the data in Table 2.

## 3. Discussion

The observation that one flower visitor taxon fragmented the pollinium of the orchid, smeared the resulting pollen fragments onto its body, and subsequently visited another flower of the same individual, where it again disintegrated the pollinium and accumulated additional pollen before departing, challenges the presumed exclusivity of autonomous self-pollination in *E. bugacensis.* This behavior directly enables geitonogamous pollen deposition and suggests the potential for xenogamous pollen transfer.

The visiting individuals were identified as halictid bees (Hymenoptera: Halictidae) based on morphological characteristics observed in the field and in photographs. Individuals were not captured for species-level identification, as capturing them would have risked disturbance to this highly protected plant species. Consequently, no microscopic analysis of pollen carried in the scopa was performed.

In contrast, our pollinator exclusion experiments demonstrated that the applied net covering exerted a stronger positive effect on reproductive traits than the accessibility of flowers to pollinators per se.

### 3.1. Evaluating the Role of Flower Visitors in Pollination

**Sweat bees (Halictidae)** are important pollinators of numerous plant species, including several *Epipactis* taxa [16,43]. They use nectar as their primary energy source for flight and activity [55,56]. Pollen represents their only solid food and is collected by adult females both for self-nutrition and the provision of brood cells for larvae [57,58,59].

Among the recorded visitors of *E. bugacensis,* only this taxon had been observed to transport pollen. During feeding on *E. bugacensis* pollen, the sweat bee fragmented the pollinium, and fragments of this disintegrated massula were deposited in large quantities onto the flower’s own stigmatic surface, while remaining pollen adhered to the body and was transferred to neighboring flowers, allowing geitonogamous pollen deposition. The individual then departed with a fully loaded scopa. This observation demonstrates the following:

1. During the observation period, *E. bugacensis* functioned as a pollen flower for the visiting sweat bee;
2. Fragmentation of the pollinium by the visiting sweat bee facilitates autogamous pollen deposition in the flowers;
3. Transferring pollinium fragments to pollen-carrying hairs and transporting them in this manner can potentially enable geitonogamy and xenogamy;
4. In orchids, pollen removal is not only possible through the intact extraction of the entire pollinarium [3,4,6,33,44,60] but also through its fragmentation and transfer via the pollinator’s body;
5. Pollen movement is not only directed toward *E. bugacensis* via heterospecific pollen donors [27,54] but this species itself may also transport its pollen to other individuals, potentially inducing xenogamy.

Although the observed mechanism was documented during three visitation events and three pollen transport events, the behavioral sequence encompassed all steps required for pollinium disaggregation, stigmatic contact, and flower-to-flower transport, thereby demonstrating the mechanistic plausibility of pollen transfer.

This phenomenon is likely not unique among orchids, including species of the genus *Epipactis*. Molnár and Csábi [60] note that “in facultative autogamous species, flower visitation may remove the pollen mass from the flower. Thus, insect pollination may occur, but fertilization may also take place with the flower’s own pollen even without this”. Although they discuss only the intact removal of the entire pollen mass, one of the three figures illustrating this statement depicts an obligately autogamous *Limodorum abortivum* with a disintegrating pollinium, on which a megachilid bee (Megachilidae) is shown bearing large amounts of pollen attached to the ventral pollen-collecting scopa of the abdomen. Rewicz et al. [49] reported that, depending on habitat, 32–39% of insects visiting *Epipactis helleborine* possessed chewing mouthparts (e.g., orthopterans, beetles) and 9–20% chewing-sucking mouthparts (bees). Similar to the halictid bees observed in our study, other insects with functional mandibles for chewing may likewise be capable of fragmenting and transporting pollinia.

It should be noted that halictid bees may also be regarded as pollen thieves, as their very thorough pollen collection could theoretically reduce the number of seeds formed within capsules.

**Mosquitoes (Culicidae)** have also been reported by others to visit various *Epipactis* species [49,54], but they were considered nectar consumers rather than effective pollinators. Based on the body position and small body size of the mosquito we observed, which clearly avoided the gynostemium, we consider it unlikely that mosquitoes transported pollinia from other *Epipactis* individuals to the studied species. It is even less likely that they could have carried fragments of *E. bugacensis* massulae, let alone entire pollinia.

**Hoverflies (Syrphidae)** are regarded as significant pollinators and are known to feed on *Epipactis* species, often transporting pollinia [35,44,49,50,51,52,61,62,63,64]. Although no direct contact between the gynostemium and either *Episyrphus balteatus*, *Platycheirus splendidus*, or unidentified individuals was observed in our study, their role as potential pollen vectors cannot be entirely excluded. While foraging for pollen or nectar, they could accidentally contact fragmented pollinia and transport pollinium fragments or bring pollinia or pollinium fragments from other individuals or species to *E. bugacensis*. Nevertheless, we consider the role of hoverflies in pollen transport of *E. bugacensis* to be negligible.

**Shield bugs (Pentatomidae)** are unlikely to participate in the pollination of *E. bugacensis*, as they showed no direct contact with the flowers, and have not been reported as pollinators of other *Epipactis* species [35,44,49,50,51,52,61,62,63,64].

A similar situation applies to **aphids (Aphididae)**, which have not been reported as pollinators of other *Epipactis* species either [35,44,49,50,51,52,61,62,63,64]. Although during heavy infestations they may approach the vicinity of pollinia, they almost certainly do not participate in pollen tetrad transport, as neither their lifestyle nor their small body size and lack of pollen-collecting hairs support such a role.

Within **ants (Formicidae)**, *Myrmica* ants have been reported as occasional flower visitors of several *Epipactis* species, including *E. albensis* [35] and *E. palustris* [34,35,43,44], while *Lasius* ants have been identified as effective pollinators of *Epipactis palustris* [44]. However, although pollen transport has been described in other *Temnothorax* species on *Neotinea maculata* and *Chenorchis singchii* [65,66], similar activity has not been documented in *Temnothorax unifasciatus*. Although ants are abundant and show high activity, their pollination efficiency is substantially reduced by frequent self- and allogrooming, poorly adapted body hairs for pollen transport, cuticular secretions that reduce pollen viability, and movement in soil, litter, and vegetation that promotes pollen abrasion [67]. As pollen forms part of their diet in addition to nectar, pollen may temporarily adhere to their bodies, allowing short-term transport [44,65]. Although we did not observe pollen feeding or transport in any of the three ant taxa recorded, their involvement in autogamy and geitonogamy cannot be excluded, nor can their potential role in reducing gamete numbers through pollen consumption.

The last arthropods observed were **spiders (Araneae)**. Individuals of *Araneus diadematus* occupied the upper parts of *E. bugacensis* inflorescences and constructed webs, but no direct interaction with pollinia or stigmatic surfaces was recorded. Spiders are therefore best interpreted as concomitant arthropods whose presence may indirectly influence pollination, either negatively by deterring insect visitors [68,69,70,71,72,73] or positively through the potential suppression of herbivores [74,75,76]. Overall, any contribution of spiders to the reproductive success of *E. bugacensis* is likely indirect rather than through direct pollen transfer.

### 3.2. Changes in Different Sexual Propagation Traits in Covered and Uncovered E. bugacensis Individuals

We explicitly interpret net covering as a compound field treatment, combining pollinator exclusion with physical protection and microhabitat modification. Under natural field conditions, pollinator exclusion cannot be fully separated from these concurrent effects; thus, the experiment reflects the ecological consequences of net covering as an integrated intervention.

Direct measurements of microclimatic variables (e.g., temperature, humidity, wind exposure) were not conducted, as repeated instrumentation or logger deployment was not feasible due to the strictly protected status of *E. bugacensis* and site-access constraints. To date, no study has quantified the joint microclimatic and pest-protection effects of pollinator-exclusion netting in orchids. Experimental and agricultural studies in other plant systems indicate that comparable netting interventions may reduce solar radiation and wind exposure, buffer temperature fluctuations, increase relative humidity, and provide a partial barrier against herbivores and pests [77,78,79,80]. These findings support the plausibility that similar mechanisms contributed to the treatment effects observed here.

Within this framework, the reproductive output of *E. bugacensis* was strongly influenced by site and covering treatment for capsule volume and seed number, but seed density remained unaffected, implying that environmental factors primarily influence total reproductive investment rather than the packing density of seeds within capsules. At the individual level, covered plants exhibited stronger and more consistent spatial relationships between reproductive traits and capsule position, whereas uncovered individuals showed reduced spatial coupling and greater variability among plants. 

#### 3.2.1. Effect of Net Covering on Fruit Set at the JF Site

In this species, the higher flower-to-capsule conversion under covering is unlikely to result from pollinator-related processes. Instead, the effect of net covering probably reflects indirect benefits, such as physical protection of buds and flowers from herbivores, pollen robbers, and mechanical damage, as well as modification of local microclimatic conditions. Enhanced humidity and reduced environmental stress around developing flowers may have improved fertilization success and early capsule development. These results highlight that in self-pollinating orchids, treatments designed to exclude pollinators may substantially influence reproductive success through non-pollination-related mechanisms.

#### 3.2.2. Interpretation of the Reproductive Trait Differences

Our results demonstrate that both capsule volume and seed number in *E. bugacensis* are strongly influenced by the interaction of locality and pollinator exclusion net-covering treatment, while seed density remains unaffected. Plants from the JF site, particularly under covered conditions, consistently produced the largest capsules and the highest seed numbers. This pattern may reflect site-specific microenvironmental differences within the poplar plantations. The covering treatment may buffer plants from desiccation and excessive sunlight, factors known to limit capsule maturation and embryo development in orchids [81,82].

The strong and consistently large effect sizes observed for capsule volume and seed number (δ > 0.50 in most contrasts) suggest that these differences are unlikely to be purely stochastic and may be biologically relevant. In contrast, the absence of significant differences in seed density may reflect greater developmental stability of this trait in *E. bugacensis*, although this interpretation remains speculative. Some orchid species adapt the volumes of their capsules and the number of seeds to the available resources under changing environmental conditions [83]. Our findings align with this pattern and suggest that *E. bugacensis* adjusts its reproductive output primarily by modulating the quantity of seeds produced rather than altering the packing density within capsules.

The comparatively poor performance of plants in the HK site may reflect less optimal habitat conditions, including more pronounced drought exposure or soil moisture limitations, which are known to reduce capsule set and seed formation in terrestrial orchids. These environmental stresses likely contribute to the smaller capsule volume and reduced seed numbers in HK populations [81,82]. This pattern has important conservation implications: populations in more marginal habitats may have reduced reproductive capacity, limiting long-term viability unless microhabitat conditions are improved or stabilized.

Taken together, the parallel trends observed in capsule volume and seed number suggest that both traits are governed by similar physiological and environmental constraints in *E. bugacensis*. The consistency of these patterns across treatments reinforces the view that local microenvironmental quality is a key determinant of reproductive output, with covering treatments potentially acting as proxies for favorable natural conditions such as canopy shading or high litter moisture.

#### 3.2.3. Spatial Patterns of Reproductive Traits Along the Flowering Shoot

The observed spatial gradients in seed number, capsule volume, and seed density along the flowering shoots of *E. bugacensis* indicate a consistent basal–apical decline in several components of reproductive output at the individual plant level. The predominance of negative correlations for seed number and capsule volume suggests that flowers located at basal positions along the inflorescence contribute disproportionately to total reproductive investment, irrespective of pollinator exclusion treatment. Such patterns are commonly interpreted as the outcome of resource allocation constraints, whereby earlier-developing basal flowers benefit from preferential access to assimilates compared with later-formed apical flowers [84,85].

The similarity in the frequency and strength of significant correlations between covered and uncovered individuals further indicates that pollinator access is unlikely to be the primary driver of the observed spatial structuring. Instead, the consistent decline in seed number and capsule volume across treatments suggests that internal resource gradients along the flowering shoot play a dominant role in shaping reproductive output. This interpretation is supported by the fact that strong linear relationships were detected in a majority of individuals in both treatments, whereas the absence of significant correlations was limited to only a few plants.

In contrast, seed density displayed considerably weaker and more variable spatial patterns, with significant correlations detected in only a small subset of individuals. This suggests that seed density may be less tightly constrained by positional effects and may be regulated independently from total seed number and capsule size. The decoupling of seed density from other reproductive traits implies that reductions in capsule volume or seed number toward apical positions do not necessarily translate into proportional changes in packing efficiency within capsules.

The occasional absence or weakening of positional correlations in some uncovered individuals may reflect local, plant-specific disturbances, such as herbivory, mechanical damage, or pathogen effects, which could disrupt otherwise regular resource allocation patterns. However, given the overall consistency of spatial trends across treatments, such deviations appear to be secondary and do not alter the general conclusion: reproductive trait variation along the flowering shoot in *E. bugacensis* is predominantly governed by internal allocation processes rather than pollinator-mediated effects.

The above-mentioned results support the view that *E. bugacensis* exhibits strong intrinsic structuring of reproductive effort along the inflorescence, with basal flowers playing a key role in seed production. Pollinator exclusion did not fundamentally modify these spatial patterns, further reinforcing the conclusion that this species relies primarily on autonomous self-pollination, with basal-apical variation in reproductive success largely driven by developmental and physiological constraints.

### 3.3. Interpretation of Mortality Differences

The observed pattern suggests that covering treatment may have offered some degree of protection, potentially reducing environmental stressors such as desiccation, extreme temperatures, or mechanical damage. Covered individuals exhibited a considerably lower mortality rate (45.5%) than those left exposed (75.8%), aligning with the hypothesis that microhabitat buffering improves survival in *E. bugacensis*.

However, the marginal statistical significance (*p* = 0.0664) indicates that the evidence is not strong enough to conclusively attribute differences in survival to the treatment. The relative risk of mortality was 1.67 (95% CI: 0.83–3.34), indicating a trend toward higher mortality in uncovered plants, although the confidence interval includes unity. The limited sample size in the covered group (*n* = 11) likely reduced statistical power. Differential survival may have modestly influenced estimates of mean reproductive output through survivorship filtering, as only surviving individuals contribute to capsule and seed measurements. Additionally, mortality in terrestrial orchids can be strongly influenced by multiple interacting environmental factors—soil moisture and microclimate [86,87,88,89], fungal symbiont availability [87,90,91], herbivory—potentially obscuring treatment effects in this dataset.

Nevertheless, the biological trend remains notable: uncovered plants experienced substantially greater mortality, suggesting that environmental exposure at the JF site may impose stress levels that covering treatments can partially mitigate. These observations align with patterns observed in other drought-sensitive terrestrial orchids, where microclimatic buffering enhances survival, especially during dry or hot periods.

### 3.4. Limitations of Our Results

#### 3.4.1. Completeness of Exclusion

Any enclosures beneath which animal presence was detected were excluded from analysis. At the HK site, no exclusion was necessary. At the JF site, organisms were detected under two nets: under one, a tiger moth caterpillar and abandoned egg shells were found on a leaf; under the other, a dead hoverfly imago was discovered, likely having entered the enclosure at the larval stage. Both cases were excluded from this study.

#### 3.4.2. Theoretical Effectiveness of Excluding Flying Insects

The enclosure mesh used could only exclude arthropods exceeding the 0.2 mm mesh aperture size in their minimum body diameter. Consequently, very small insects capable of passing through the mesh—such as some thrips species—may not have been completely excluded. Because thrips have been shown to play a key role in the pollination of Japanese *E. thunbergii* [16], their potential involvement in pollen transport of *E. bugacensis* cannot be entirely ruled out, although thrips were not observed during our study.

#### 3.4.3. Theoretical Effectiveness of Excluding Non-Flying Insects

Ants could certainly not be excluded by using this method, as they could burrow through loose sandy soil and enter or exit beneath the nets, even though this behavior was not observed. Nevertheless, the lack of specialized pollen-collecting hairs, frequent grooming behavior, reduced pollen viability due to cuticular secretions, and abrasion during movement through soil and litter—the traits outlined in Section 3.1—all argue against their involvement in xenogamy. However, their unobstructed access may have facilitated autogamy or geitonogamy, and partial restriction of their movement could have reduced these processes.

Taken together, these methodological constraints should be considered when interpreting the ecological and reproductive implications of our findings. As genetic paternity analyses were not performed, the occurrence of true xenogamy or bastardogamy cannot be confirmed and remains inferred solely from the observed pollen transfer mechanisms.

Nevertheless, beyond these limitations, our observations provide mechanistic insights into pollen movement in orchids. Together, these findings indicate that pollen removal from orchid flowers is not necessarily restricted to the intact extraction of the entire pollinarium. Pollinium fragmentation mediated by flower visitors may represent an alternative pathway for pollen movement. Consequently, the use of the term “obligate autogamy” for self-compatible, open-flowered orchid species should be applied with caution, particularly when such classifications are based primarily on floral morphology.

## 4. Materials and Methods

### 4.1. Species Overview

Latin name: *E. bugacensis* Robatsch 1990

Syn.: *Epipactis rhodanensis* Gévaudan & Robatsch

Conservation status in Hungary: Strictly protected; conservation value: 250,000 HUFIUCN Red List category: Not Evaluated (NE)

Description:

A species characterized by the production of slender, finely mealy-pubescent flowering shoots that occur singly or, less frequently, in small groups, and reach (12)–20–50(–77) cm in height. Plants are light green in Hungary; green in Germany and Austria, often with a slight violet (bronze-like) tinge; and almost always distinctly violet in France. The leaves are 3.5–5.5(–7) cm long and 2–2.5 cm wide, elliptic to lanceolate, with an acuminate apex, in numbers of (2)–3–4(–7). The bracts measure 10–24 mm in length and are shorter than the flowers. The inflorescence is a loose raceme with (2)–5–20(–50) flowers.

Flowers are slightly nodding. The sepals are light green, (7.5)–8–9(–10) mm long and 3–4 mm wide. The petals are pale greenish white, often flushed with pink. The hypochile of the lip is reddish-brown inside and nectar-producing; the mesochile is very narrow (in Hungary, its characteristics may vary by region). The epichile is a broad triangle, 4 mm wide and 4 mm long, pale pinkish and white, with pink coloration between the basal swellings. The viscidium remains visible and functional briefly after anthesis, appearing whitish, but it browns rapidly, after which pollen masses (pollinia) can no longer be removed. According to the literature, the flowers are obligately self-pollinating [19,60].

The capsule-like pseudofruits (“capsules”) are nodding, (5.5)–9–12.5(–14) mm long and (3)–5–7.5(–9) mm wide, glabrous, and occasionally with hairs only along the edges [19].

Phenology:

Vegetative emergence: April

Flowering: (May)–June–(July)

Fruit maturation: July–August

**Figure 7:**
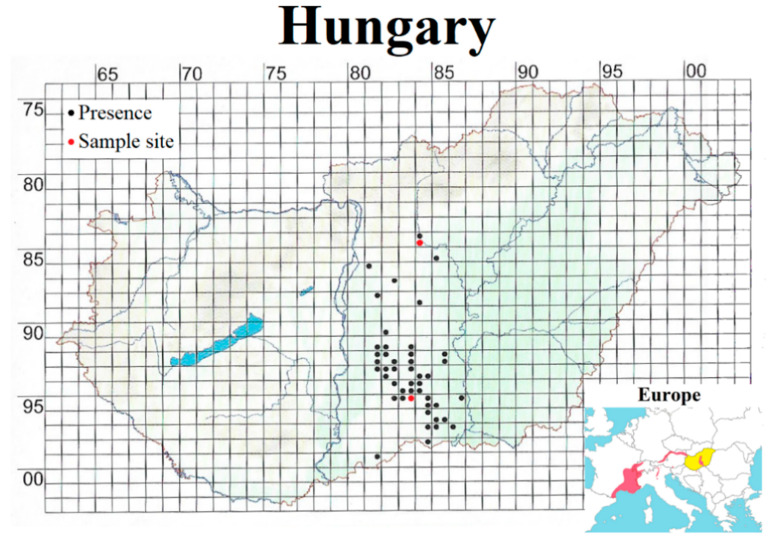
Distribution of *E. bugacensis* in Hungary [60] and Europe [92]. On the Hungarian map, red dots mark the JF (north) and HK (south) sites within the Central European flora mapping grid [94]. Basemaps [60,92] modified by the authors.

Distribution and habitat:

The precise distribution is not fully known. In Hungary, it occurs on the Danube–Tisza Interfluve (Figure 7). Additional records exist from southern and eastern France, western Switzerland, the western and northern regions of Italy, and the Austrian–German border area as well as northern Austria at elevations ranging from 0 to 1600 m.

Based on current records, the species has a lowland distribution and is moderately shade-tolerant. In Hungary, it primarily inhabits calcareous sandy soils, occurring in planted and semi-natural forests: in oak woodlands and gallery forests and beneath isolated oaks or black pines. It is most frequently found in planted poplar stands where the groundwater table lies relatively close to the surface [19,60,92].

### 4.2. Study Sites

Field research was conducted at two sampling locations: one near the town of Jászfényszaru (JF) and another approximately 125 km to the south (in a straight line) near the village of Harkakötöny (HK). Both sites are poplar plantations (*Populus × euramericana*) established on calcareous sandy soils and situated within the Central European floristic mapping grid squares 84,843 and 95,832, respectively (Figure 7). Each locality is surrounded by arable fields and similarly non-native tree plantations. The HK site was partially adjacent to dry sandy grasslands. The herb layer at both sites is extremely species-poor and sparse in individuals; aside from one or two grass species (e.g., *Brachypodium sylvaticum*, *Bromus tectorum*, *Bromus sterilis*, *Dactylis glomerata*) and a few common dicots (*Geum urbanum*, *Glechoma hederacea*) as well as the frequently abundant invasive *Asclepias syriaca*, the ground layer is largely bare. Within these seemingly competition-free environments, shoots of *E. bugacensis* occur scattered individually or in small patches.

The climate of the Jászfényszaru sampling area is moderately warm and dry, with an annual mean sunshine duration of approximately 1980 h. The annual mean temperature ranges between 10.1–10.3 °C, while the average during the growing season ranges between 17.0–17.5 °C. Frost-free conditions typically occur between 8 April and 28 October. Annual precipitation totals 520–560 mm, of which 300–320 mm falls during the growing season. The Harkakötöny sampling area has a somewhat warmer and more humid climate, with an annual mean sunshine duration of around 2030 h. The annual mean temperature ranges between 10.5–10.7 °C, and that of the growing season is approximately 17.5 °C. The frost-free period generally lasts from 1 April to 25 October. Annual precipitation totals 550–580 mm, with 310–360 mm occurring during the growing season [93].

### 4.3. Field Methods

#### 4.3.1. Observation of Visitors

Floral visitor observations were restricted to the JF site to ensure repeated and temporally intensive monitoring during peak flowering, which was not feasible at the HK site. Observations were carried out personally by direct visual inspection with the naked eye and supported by photographic documentation. When possible, and without harming individuals of the strictly protected plant species, flower visitor collection was also performed.

Systematic observations were conducted in 2019, spanning eight days within the 15-day flowering period from 19 June to 3 July. The first day of flowering was defined as the day when the first bud opened on any of those 13 individuals that could be continuously observed from a single fixed position, while the last day was defined as the day when the petals of the final flower had completely withered. On each observation day, at least two continuous hours were spent observing between 08:00 and 18:00, most commonly between 08:00 and 14:00.

During systematic observations, the following parameters were recorded: date, weather conditions (cloud cover, precipitation, temperature), duration of observation, identity and behavior of potential pollinators, and the method of data collection (photography or capture).

Cloud cover was classified into the following categories:

Clear/sunny (S): 0/8–1/8 of the sky covered by clouds;

Slightly cloudy (SC): 2/8–3/8 cloud cover;

Partly cloudy/moderately cloudy (PC): 4/8–5/8 cloud cover;

Mostly cloudy (MC): 6/8–7/8 cloud cover;

Overcast (O): 8/8 cloud cover, no cloud-free areas.

Precipitation was recorded as present (+) or absent (−) during the observation period. Air temperature was measured in °C using a liquid-in-glass thermometer placed at the study site.

Observed visitors were identified to the lowest reliable taxonomic level. Photographs were taken using a Pentax K10D camera (Pentax Corporation, Tokyo, Japan) equipped with a Pentax 100 mm 1:1 macro lens (Pentax Corporation, Tokyo, Japan), both handheld and tripod-mounted, using the highest possible shutter speed.

Flower visitor collection was performed using an insect net. Collected insects were preserved in vials containing 70% (*v*/*v*) ethanol and identified within the same week.

In addition to systematic monitoring of the 13 focal individuals, supplementary opportunistic observations of flower visitors were conducted during separate field visits on 4 June 2018 (10:00–12:00), 12 June 2022 (17:00–18:00, bud stage), and 23 June 2022 (10:00–12:00). These observations were independent of the fixed observation protocol and served to document additional visitation events under natural field conditions. All visits took place under undisturbed sunny and warm conditions at temperatures between 26 and 28 °C and were documented using field notes and photographs.

#### 4.3.2. Assessment of Pollinator Dependence of Fruit and Seed Set

##### Field Experiments

Under natural field conditions, net-covering applied to exclude pollinators also provides physical protection to the covered plant and modifies the microhabitat’s environmental factors under the net; therefore, the experiment examines the net-covering intervention as a whole, not just the exclusion of pollinators.

At both study sites, individual plants were selected sequentially in the order of encounter along the transects. To assign treatments, every third individual was designated for the covered treatment, while the remaining individuals were left uncovered. This systematic allocation ensured an unbiased distribution of treatments without subjective selection. At the JF site, five transects located 20–50 m apart were established within the plantation stand. At the HK site, four transects were used. All of them were situated in separate plantations, more than 50 m apart. Individuals were selected sequentially within each transect, and treatment allocation was applied within transects. The transects were established within structurally homogeneous, even-aged (21–25 years old) poplar plantation stands located on flat terrain. No visible microtopographic variation occurred within sampling areas, and canopy closure (approx. 90–95%), litter depth (3–5 cm), and soil surface conditions appeared spatially uniform along transects. Therefore, systematic treatment allocation was not associated with detectable microhabitat gradients in light availability, litter accumulation, or soil moisture conditions. At the JF site, the number of uncovered individuals was intentionally increased to enhance sample size and statistical power for comparisons involving reproductive output. Nets were inspected daily at the Jászfényszaru (JF) site throughout the flowering period. At the Harkakötöny (HK) site, nets were inspected every 3–4 days due to logistical constraints. No non-target arthropods were detected beneath nets at the HK site during these inspections. At the JF site, two covered individuals were found to harbor non-target organisms beneath the netting and were excluded from subsequent analyses to ensure treatment integrity. As a result, the final sample sizes were 12 covered and 24 uncovered individuals at the HK site, and 11 covered and 91 uncovered individuals at the JF site. Because *E. bugacensis* is a strictly protected species with naturally low population sizes, sample sizes were necessarily limited to minimize disturbance.

Covering was applied before bud opening on 11 June 2022 at the JF site and on 12 June 2022 at the HK site. The mesh covers were removed shortly before capsule dehiscence, while the capsules were still closed: on 26 July 2022 at the JF site and 22 July 2022 at the HK site. At the JF site, fruit set and plant mortality were again recorded for each individual.

At both study sites, numbered, thin wooden or reed sticks were placed next to each studied individual. At each location, a subset of *E. bugacensis* individuals was excluded from potential pollinator access, while the remaining individuals remained accessible to them.

Pollinator exclusion was achieved using 30 × 45 cm, 100% polyester recyclable food-storage mesh bags (SPAR Magyarország Kereskedelmi Kft., Bicske, Hungary; product origin: China) with a mesh size of 0.2 mm. The mesh did not contact the plants and allowed sufficient air circulation. Supporting frames were constructed from two 130 cm stainless-steel wires bent into U shapes. One wire was fitted with a small single-loop twist at its midpoint, through which the second U-shaped wire was passed. Before installation, the lower 10 cm of each wire end was bent outward at a 20–30° angle to ensure penetration into the soil away from the plant base, preventing damage to rhizomes or roots.

The four ends of the assembled frame were inserted into the soil around the plant, forming the corners of an imaginary square (approx. 8–12 cm side length). The mesh bag was then pulled over the frame and secured at the base using its drawstring. Leaf litter and soil were placed around the lower edge to prevent insect entry from below, ensuring effective pollinator exclusion. Net-covering may also alter local microclimatic conditions and provide physical protection, which should be considered when interpreting treatment effects.

Immediately before covering, the number of buds on each examined individual was counted and recorded. To verify baseline comparability between treatments at the JF site, pre-treatment bud/flower numbers were compared between covered and uncovered individuals using a Mann–Whitney U test. No significant difference was detected (covered: median = 9, IQR = 4.5–15.0, n = 11; uncovered: median = 7, IQR = 4.0–11.0, n = 91; U = 603.5; *p* = 0.268), indicating similar initial reproductive potential prior to net application. Plants were monitored every two days until the opening of the first flower (17 June), and mortality before flowering was recorded. These bud number and mortality assessments were restricted to the JF site to ensure standardized, observer-consistent measurements during the observation period.

At both sites, at least two intact capsules were collected from each surviving individual when available. At the JF site, all capsules were collected from four covered individuals (Plant IDs: 0, 8, 15, and 40) and seven uncovered individuals (Plant IDs: 31, 51, 63, 65, 67, 74, and 76). This allowed us to assess, at the individual level, whether capsule position within the inflorescence was correlated with seed number. Each capsule was placed into a uniquely numbered envelope and transported to the laboratory.

In the laboratory, total capsule length (L) and maximum capsule width (W) were measured to the nearest millimeter. Capsule volume was approximated as a prolate spheroid (ovoid) using the formula

V = (π/6) × L × W^2^,

which is equivalent to the ellipsoid equation V = (4/3)πa^2^b, where a = W/2 and b = L/2. Seeds were counted for each capsule.

Seed counting was carried out individually under a stereomicroscope. Seeds were carefully transferred into a small tray made of graph paper, evenly distributed with a dissecting needle, and counted. Counting was repeated until two consecutive counts yielded identical totals. Due to the extremely low mass of orchid seeds, masks were worn during handling to prevent displacement caused by breathing. Seed count data were recorded in Microsoft Excel files by site, individual, and capsule. Alternative estimation approaches reported in the literature [95,96,97,98] were explored, but did not provide sufficient accuracy for this dataset.

After completion of the study, seeds were returned to their original collection sites and dispersed in situ in September.

#### 4.3.3. Data Preparation

Datasets from both locations were compiled into a unified structure with four treatment groups (JF covered, JF uncovered, HK covered, HK uncovered). All recorded values were retained in the analyses, as even extreme observations were considered to reflect real biological variation rather than measurement artefacts. Sensitivity analyses excluding values beyond 1.5× the interquartile range did not alter the direction or statistical significance of the observed treatment effects.

All statistical analyses and data visualizations were performed using Python 3.11 (Python Software Foundation, Wilmington, DE, USA) with the SciPy 1.11, NumPy 1.26, pandas 2.1, and Matplotlib 3.8 libraries, and Microsoft Excel (Microsoft 365, Microsoft Corporation, Redmond, WA, USA).

#### 4.3.4. Statistical Analysis

##### Fertilization Success

At the JF site, fruit set was evaluated at the flower level to test whether pollinator exclusion affected the probability of capsule formation. For covered and uncovered individuals, the total number of flowers and the number of developed capsules were recorded, and fruit set was expressed as the proportion of flowers producing capsules. Differences in fruit set between treatments were tested using Fisher’s Exact Test, which is appropriate for comparing proportions in contingency tables, especially when sample sizes are unbalanced. In addition, to account for the nesting of flowers within plants, we fitted a binomial generalized linear mixed model (GLMM) with treatment as a fixed effect and plant identity as a random intercept; overdispersion was assessed and an observation-level random effect was considered if needed. Effect sizes are reported as odds ratios with 95% confidence intervals.

To determine whether there were statistically significant differences between the sizes of capsules (cm^3^), the seed numbers of capsules, and the seed density of capsules (seed/cm^3^) of covered and uncovered individuals at the JF and HK site, the following statistical procedures were applied. Normality was tested using the Shapiro–Wilk test and homogeneity of variances using Levene’s test. Because assumptions of parametric ANOVA were violated, non-parametric analyses were applied. Differences among the four groups were evaluated using the Kruskal–Wallis test, followed by Dunn’s post hoc test with Holm correction. Specifically, for the standard dataset (two lowest capsules per individual at both sites), mean capsule volume, mean seed number per capsule, and mean seed density were calculated per plant and compared across the four sites × treatment groups. As a robustness check, we also fitted linear mixed-effects models at the capsule level with plant identity as a random intercept and fixed effects for site, treatment, and their interaction. Effect sizes were calculated using Cliff’s delta (δ). Graphs were generated as boxplots. Significance markers corresponding to adjusted pairwise *p*-values (*p* < 0.05 = L (low); *p* < 0.01 = M (medium); *p* < 0.001 = H (high)) were included in all figures.

##### Analysis of Correlations Between Capsule Position and Reproductive Traits

Correlation analyses between capsule position and reproductive traits were conducted for ten individuals (four covered and six uncovered), for which seed number (pieces), capsule volume (cm^3^), and seed density (seed number/cm^3^) could be consistently quantified.

Relationships between capsule position and seed number were assessed using Pearson correlation analysis for covered and uncovered individuals at the JF site. In addition, to evaluate population-level trends while accounting for between-plant heterogeneity, linear mixed-effects models were fitted with capsule position as a fixed effect and plant identity included as a random intercept and random slope. Results were visualized using scatter plots with fitted linear trend lines and coefficients of determination (R^2^). Statistical significance was evaluated at *p* < 0.01.

For each individual, sample size (*n*) corresponded to the number of capsules included in the analysis.

##### Mortality Analysis

Mortality rates of *E. bugacensis* individuals at the JF site were analyzed to determine whether covering treatment influenced survival. The test was implemented in Python using Fisher’s Exact Test due to the small sample size in the covered group, which returned the exact probability of observing the given contingency table under the null hypothesis of equal mortality proportions across treatments.

## 5. Conclusions

Our study confirms that *E. bugacensis* is predominantly self-pollinating; however, the present results indicate that its reproductive strategy cannot be regarded as strictly obligate autogamy. While autonomous self-pollination clearly represents the dominant mode of reproduction, our field observations demonstrate that limited, insect-mediated pollen redistribution may occasionally occur.

In particular, we document a previously unreported pollination-related mechanism in *E. bugacensis*, whereby halictid bees fragment pollinia and transfer pollen grains directly onto stigmatic surfaces. This process fundamentally differs from classical pollinarium-mediated pollen transfer and represents an alternative pathway of pollen deposition. Through pollinium fragmentation, pollen may be redistributed within a single flower, among flowers of the same individual, or potentially between neighboring individuals. As a result, *E. bugacensis* may function not only as a pollen acceptor but also as a pollen donor, thereby allowing rare instances of geitonogamy and potentially enabling xenogamous or bastardogamous pollen deposition in addition to prevalent autogamy. The observed behavior further suggests that flowers of *E. bugacensis* serve primarily as a pollen resource rather than a nectar source for halictid bees.

Despite these occasional pollen transfer events, our experimental results indicate that the effects of net covering on reproductive success were substantially stronger than those attributable to pollinator access. Increased fruit set, capsule volume, and seed number under covering treatments are best explained by indirect benefits such as physical protection and microclimatic buffering, including reduced desiccation stress and mechanical damage. Similar interactions between microhabitat quality, plant development, and reproductive output have been widely documented under changing climatic conditions and increasing environmental stress [89,91,99,100].

Spatial patterns along flowering shoots further revealed strong intrinsic constraints on reproductive allocation, with basal flowers contributing disproportionately to seed production, while seed density remained largely invariant across sites and treatments. Such canalization of reproductive traits under variable resource availability has been reported in both wild and cultivated plant species and is considered an important mechanism of reproductive stability [49,101,102,103,104,105].

Taken together, our findings indicate that the reproductive success of *E. bugacensis* is governed predominantly by microhabitat quality and internal developmental processes, while pollinators play a limited and context-dependent role. Under ongoing climate change and decreasing habitat heterogeneity, the preservation of favorable small-scale environmental conditions may therefore be as critical for population persistence as pollinator availability itself, particularly in self-pollinating plant species [89,106,107,108,109,110,111,112,113].

## Figures and Tables

**Table 1:** The date(s), time(s), weather conditions, and behavior of the observed visitor(s) on the orchid during the observations, the interactions with reproductive structures, and the data recording method as evidence.

Taxon	Date(s)	Time	Weather	Floral Contact	Behavior	Evidence
Halictidae	4 June 2018	10:00–12:00	Sunny, 26–27 °C	Sepals, petals, hypochil, gynostemium, pollinia	Pollinium shredding. Collection and transport of its fragments.	Photo + Field diary
Culicidae	4 June 2018	10:00–12:00	Sunny, 26–27 °C	Lip only	Nectar probing, no reproductive contact	Field diary
*Platycheirus splendidus*	19 June 2019	08:00–12:00	Semi-cloudy, 27–30 °C	No	Hovering, repeated inspections only	Capture + Field diary
Syrphidae (cf. *Platycheirus splendidus*)	24 June 2019	14:00–18:30	Sunny, 28–30 °C	No	Hovering, repeated inspections only	Field diary
Syrphidae (cf. *Episyrphus balteatus*)	4 June 2018	10:00–12:00	Sunny, 26–27 °C	No	Hovering, repeated inspections only	Photo + Field diary
*Episyrphus balteatus*	23 June 2022	10:00–12:00	Sunny, 28–30 °C	Sepal peaks only	Brief landings, no contact with the gynostemium	Photo + Field diary
Pentatomidae	20 June 2019; 23 June 2022	10:00–13:30	Sunny/SC, 27–30 °C	No	Resting; capsule feeding	Photo + Collection + Field diary
Aphididae	3 July 2019; 12 June 2022	08:00–18:00	PC/S, 25–30 °C	Petal surfaces only	Colonies on budding shoots	Photo + Field diary
*Temnothorax unifasciatus*	27 June 2019; 3 July 2019	08:00–14:00	PC/MC, 25–30 °C	Sepals, petals, hypochil, gynostemium, pollinia	Intensive searching in the flower	Photo + Collection + Field diary
*Myrmica* sp.	20 June 2020	10:00–13:30	Semi-cloudy, 27–30 °C	Sepals, petals, hypochil, gynostemium, pollinia	Intensive searching in the flower	Photo + Field diary
*Lasius* sp.	12 June 2022	16:00–18:00	Sunny, 28–30 °C	No	Aphid tending on budding stem	Photo + Field diary
Araneae	29 June 2019	11:00–14:00	Sunny, 28–30 °C	Sepal peaks only	Web construction	Field diary
*Araneus diadematus*	23 June 2022	10:00–12:00	Sunny, 28–30 °C	Sepal peaks only	Web construction	Photo + Field diary

**Table 2:** Mortality outcomes of *E. bugacensis* individuals under covered and uncovered treatments at the JF study site. Numbers of dead and surviving individuals are given with percentages relative to the total number of plants per treatment.

Treatment	Dead Individual (%)	Alive Individual (%)	Total Individual (%)
Covered	5 (45.5%)	6 (54.5%)	11 (100%)
Uncovered	69 (75.8%)	22 (24.2%)	91 (100%)

## Data Availability

The data presented in this study are available from the corresponding author upon reasonable request.

## Abbreviations

The following abbreviations are used in this manuscript:

JF	Jászfényszaru (town)
HK	Harkakötöny (village)
*E.*	*Epipactis*
